# Comparative Transcriptome and Metabolome Analysis of Resistant and Susceptible *Piper* Species Upon Infection by the Oomycete *Phytophthora Capsici*

**DOI:** 10.3389/fpls.2022.864927

**Published:** 2022-06-23

**Authors:** Rui Fan, Xiao-yuan Tao, Zhi-qiang Xia, Soonliang Sim, Li-song Hu, Bao-duo Wu, Qing-huang Wang, Chao-yun Hao

**Affiliations:** ^1^Spice and Beverage Research Institute, Chinese Academy of Tropical Agricultural Sciences (CATAS), Wanning, China; ^2^Central Laboratory, State Key Laboratory for Managing Biotic and Chemical Threats to the Quality and Safety of Agro-Products, Zhejiang Academy of Agricultural Sciences, Hangzhou, China; ^3^Hainan University, Haikou, China; ^4^Academy of Sciences Malaysia, Kuala Lumpur, Malaysia; ^5^Key Laboratory of Genetic Resources Utilization of Spice and Beverage Crops, Ministry of Agriculture and Rural Affairs, Wanning, China; ^6^Hainan Provincial Key Laboratory of Genetic Improvement and Quality Regulation for Tropical Spice and Beverage Crops, Wanning, China

**Keywords:** black pepper, *Phytophthora capsici*, pathogen defense, phytohormones, phenylpropanoid

## Abstract

*Phytophthora capsici* is a destructive oomycete pathogen that causes devastating disease in black pepper, resulting in a significant decline in yield and economic losses. *Piper nigrum* (black pepper) is documented as susceptible to *P. capsici*, whereas its close relative *Piper flaviflorum* is known to be resistant. However, the molecular mechanism underlying the resistance of *P. flaviflorum* remains obscure. In this study, we conducted a comparative transcriptome and metabolome analysis between *P. flaviflorum* and *P. nigrum* upon *P. capsici* infection and found substantial differences in their gene expression profiles, with altered genes being significantly enriched in terms relating to plant-pathogen interaction, phytohormone signal transduction, and secondary metabolic pathways, including phenylpropanoid biosynthesis. Further metabolome analysis revealed the resistant *P. flaviflorum* to have a high background endogenous ABA reservoir and time-course-dependent accumulation of ABA and SA upon *P. capsici* inoculation, while the susceptible *P. nigrum* had a high background endogenous IAA reservoir and time-course-dependent accumulation of JA-Ile, the active form of JA. Investigation of the phenylpropanoid biosynthesis metabolome further indicated the resistant *P. flaviflorum* to have more accumulation of lignin precursors than the susceptible *P. nigrum*, resulting in a higher accumulation after inoculation. This study provides an overall characterization of biologically important pathways underlying the resistance of *P. flaviflorum*, which theoretically explains the advantage of using this species as rootstock for the management of oomycete pathogen in black pepper production.

## Introduction

Pepper (*Piper nigrum* L.), the “King of Spices,” is generally called black pepper to distinguish it from the chili pepper and sweet pepper (*Capsicum annuum L*.). The dried fruits and seeds of *P. nigrum* are, respectively, marketed as black and white peppercorns. Both feature a characteristic pungency and distinct aroma, and hence they are used as ingredients in food. Additionally, the extract from peppercorns is used in the food processing industry as a preservative for meat and sausage. Furthermore, pepper plant parts, particularly the roots, have well-documented curative and restorative powers and are used in traditional medicines in many parts of the world (Fan et al., 2020). In keeping with its utility, *P. nigrum*, which originated in South India, is currently planted in many tropical and subtropical regions in Asia, Africa, and Latin America. The major pepper-producing countries are Vietnam, India, Indonesia, Brazil, China, and Malaysia. Black pepper cultivation faces many constraints, including pests and diseases, of which the most devastating is Phytophthora foot-rot caused by the oomycete *Phytophthora capsici*. This disease takes a heavy toll in all pepper-growing countries and can cause total loss in individual holdings. Thus, the development of an effective measure for the control of this disease is highly desirable, including the breeding of a Phytophthora foot-rot-resistant variety.

*Phytophthora capsici* also infects cucurbits, chili peppers, and tomatoes (Leonian, 1922; Ding et al., 2015). To date, in-depth research on plant resistance to *Phytophthora capsici* has been carried out in members of the family Solanaceae, and progress has been made in the study of *P. capsici*-plant interactions, focusing specifically on effector-triggered immunity (ETI), pattern-triggered immunity (PTI), transcription factors (TFs), phytohormone signaling, and secondary metabolism (Liu et al., 2014). *Phytophthora* pathogens have been found to secrete a significant number of cytoplasmic effectors, including RXLR and CRN effectors, to evade host resistance, and many *Phytophthora* RXLR effectors have been shown to modulate plant immunity in order to promote infection. For example, *PexRD2*, a *Phytophthora infestans* effector, inhibits potato host immunity by targeting MAPKKKε (King et al., 2014). The *Phytophthora sojae* effector *PsAvh262* stabilizes ER-luminal binding immunoglobulin proteins (BiPs) and prevents cell death induced by ER stress (Jing et al., 2016). Similarly, the *P. capsici* RXLR effector *PcAvr3a12* targets the FK506-binding protein FKBP15-2 to suppress ER stress-mediated plant immunity. The ethylene signaling pathway is also crucial in plant responses to biological stress induced by *P. capsici*; for example, the ethylene-responsive factor *CaPTI1* significantly attenuates the defense response in pepper by reducing the expression of multiple defense-related genes, and *CaAP2/ERF064* can actively regulate the resistance response by regulating the transcription of pathogenesis-related genes (Jin et al., 2019). Also implicated as negative regulators of defense responses under *P. capsici* infection in chili pepper (*Capsicum annuum*) are members of the SBP-box plant-specific transcription factor family, including *CaSBP08, CaSBP11, CaSBP12*, and *CaSBP1* (Zhang et al., 2020). Meanwhile, xyloglucan-specific endoglucanase inhibitor proteins (XeGIPs) have been found to inhibit the specific endo-1,4-glucanase produced by *P. capsici* and to attack xyloglucan linkages in plant cell walls (Yoshizawa et al., 2012). Species resistant to *P. capsici* have been shown to respond to colonization by inducing a severe basal defense response and production of phytoalexins, including phenylpropanoid substances and terpenoids (Cui et al., 2000), of which the lignin and cinnamaldehyde taken from its branches particularly contribute to *Phytophthora* resistance.

Control measures for Phytophthora foot-rot have been a subject of study for many years; such measures include breeding and selection of resistant varieties, grafting using resistant *Piper* species as the rootstock, chemical treatment, crop rotation, soil disinfection, and application of autogenous bacterins on the rhizosphere (Hao et al., 2016). However, no Phytophthora foot-rot disease-resistant varieties have yet been successfully bred. Disease management has mainly relied on the application of fungicides, which are toxic, pollute the environment, increase production cost, and yet are not very effective. The natural genetic diversity of resistance traits represents a useful resource for understanding resistance mechanisms and their use in resistance breeding programs. In China, *Piper nigrum* cv. *Reyin-1*, a high-yielding black pepper cultivar selected by the Chinese Academy of Tropical Agricultural Sciences (Hu et al., 2019), is planted in more than 90% of the black pepper growing area. However, this cultivar is susceptible to *P. capsici*. Meanwhile, a wild species*, Piper flaviflorum*, found in the Yunnan and Hainan provinces of China, has been reported to be highly resistant to Phytophthora foot-rot disease (Gordo et al., 2012). Understanding the differences in *P. capsici-*plant interactions between susceptible and resistant species of *Piper* and the molecular mechanisms underlying the resistant phenotype are key in successfully breeding resistant varieties of black pepper.

Due to the lack of a reference genome, previous research was unable to establish the transcriptome dynamics in play throughout the defensive response of black pepper infected by *Phytophthora* (Hao et al., 2016). However, with the advent of genome sequencing and deep-sequencing technologies, studying the resistance mechanisms of black pepper is now possible (Hu et al., 2019). Thus, we performed a comparative transcriptome and metabolome analysis of susceptible and resistant *Piper* species upon *P. capsici* infection based on the time-course sampling of roots. We identified nine gene clusters as having significantly different expression profiles between the susceptible *P. nigrum* and the resistant *P. flaviflorum*. Based on these differentially expressed clusters, we investigated in more detail the processes of plant-pathogen interaction, phytohormone signaling and response, and lignin biosynthesis. We further illustrated the co-network of TFs, phytohormones, and metabolic pathways in *Piper* species that are modulated in response to infection by the pathogen *P. capsici*.

## Methods

### Plant and Pathogen Materials

Five-node cuttings from 1-year-old plants of *P. nigrum* and *P. flaviflorum* grown in the *Piper* species Germplasm Repository were taken for rooting. Those rooted cuttings were used as the experimental materials in this study. The pathogen *P. capsici* was incubated for 7 days on potato dextrose agar plates. Immediately before inoculation, the midpoint of the third internode, as counted from the tip of a rooted cutting, was injured with a syringe needle. A 3-mm inoculating disk taken from the growing margin of *P. capsici* was then patched onto the injured point and covered with a wet cotton pad to prevent drying. The pad was tied onto the stem with a polyethylene strip to maintain the position of the inoculating disk. Inoculated plants were incubated at a constant temperature of 25–28°C for 0, 4, 12, 24, and 48 h in a greenhouse with 75–90% relative humidity. Plants in the control group were injured in the same manner as described above, but instead of the agar disk, distilled water was applied to the injured point and then covered. Three root samples of each of the two species of *Piper* were collected at each of the five time points. All samples were frozen immediately in liquid nitrogen, and later freeze-dried and stored at −80°C until used for RNA-seq, determination of chemical molecule contents, and qPCR. Three replicates of stem samples were also collected from the plants for lignin content determination.

### RNA-Seq

TRIzol reagent (Qiangen, China) was used for the extraction of total RNA from the frozen stem and root samples. Quantification of total RNA was carried out using a Nanodrop 2000. RNA sample preparation used 3 μg of RNA per sample as the input material. The NEBNext Ultra RNA Library Prep Kit for Illumina (NEB, USA) was used to generate sequencing libraries according to the manufacturer's instructions, and index codes were added to enable the attribution of sequences to each sample. Sequencing of cDNA libraries was performed on an Illumina HiSeq X-10 by Novogene (http://www.novogene.com/).

The RNA-seq data were analyzed using HISAT (hierarchical indexing for spliced alignment of transcripts), StringTie, and Ballgown (Pertea et al., 2016). Clean reads were subsequently mapped to the *Piper nigrum* genome provided by the NCBI (PRJNA529758) with Bowtie2 (Langmead, 2009). Transcript expression levels were estimated by adopting the transcripts per million (TPM) approach (Mortazavi et al., 2008).

### Analysis of Gene Expression Profiles

Gene expression profiles were derived from normalized time-course RNA-seq data and compared between resistant (*P. flaviflorum*) and susceptible (*P. nigrum*) species of *Piper* with the *maSigPro* package, implemented in the R software environment (http://www.r-project.org/), following the user's guide with settings of Q = 0.05, rsq = 0.5 (Conesa et al., 2006). The genes identified as having significantly different expressions were graphically presented by plotting gene expression profiles and grouped in different clusters. Each cluster was subjected to Gene Ontology (GO) enrichment analysis, and the results were visualized as word clouds generated by the WocEA software (Ning et al., 2018).

### Determination of Phytohormones

For the detection of phytohormones, sampled tissue (50 mg) was extracted with a solution of 15:4:1 MeOH:water:formic acid. Before LC-MS/MS analysis, the combined extracts were dried under nitrogen gas stream, reconstituted in 80% methanol (V/V), and filtrated (PTFE, 0.22 μm; Anpel). Phytohormones were detected by a standard method from MetWare Ltd. (http://www.metware.cn/, Wuhan, China) using the AB Sciex QTRAP 6500 LC-MS/MS platform. Each assay was performed in triplicate; the 26 plant hormone standards used in the assays were purchased from Olchemim Ltd. (Olomouc, Czech Republic) and Sigma (St. Louis, MO, USA).

### Detection of Lignin Precursors

After burnisher treatment, 100 mg of powdered sample was transferred into 0.6 ml of extraction solution and stored overnight at 4°C. Subsequently, the reactions were centrifuged at 10,000 × *g* for 10 min, and the supernatants were filtered by a membrane filter (0.22 μm); filtrates were then used for content analyses, conducted using tandem mass spectrometry (MS/MS, Applied Biosystems 4500 QTRAP) and ultra-performance liquid photography [UPLC, Shim-pack UFLC SHIMADZU CBM30A equipped with a Waters ACQUITY UPLC HSS T3 C18 (1.8 μm, 2.1 ×100 mm)] with the absorbance of the eluate measured at 254 nm. In UPLC analyses, Solvent A was 0.04% acetic acid (LC/MS grade) in water (HPLC grade) and Solvent B was 0.04% acetic acid in acetonitrile (OmniSolv, HPLC grade); the solvent gradient parameters were as follows: starting conditions of 95% A, 5% B. Within 10 min, a linear gradient to 5% A, 95% B was programmed and was kept for 1 min. Subsequently, a composition of 95% A, 5% B was adjusted within 0.10 min and kept for 2.9 min. A volume of 4 μl of the extracted material was injected at 40°C, and the flow rate was 0.35 ml/min. The MS parameters were as follows: electrospray ionization temperature 550°C, voltage 5,500 V, and curtain gas 30 psi. The collision gas (CAD) was high. The determination and the methods of analysis had been established in a previous study (Chen et al., 2013). A total of fourteen molecules involved in the lignin pathway were quantified as follows: sinapinaldehyde, p-coumaraldehyde, L-phenylalanine, coniferyl alcohol, sinapic acid, caffeate, p-coumaric acid, sinapyl alcohol, ferulic acid, 4-hydroxy-3-methoxycinnamaldehyde, p-coumaryl alcohol, caffeyl aldehyde, caffeyl alcohol, and cinnamic acid.

### GC/MS Content Determination for Lignin and Its Derived Monomers

In total, 5 g of stem sample from each of the two *Piper* species was fully ground with liquid nitrogen and combined with a fresh reaction solution (2.5% BF_3_ and 10% EtSH dissolved in dioxane), then stewed for 4 h at 100°C. Reactions were terminated by 5 min incubation at −20°C. Lignocerane (0.1 mg/ml) was used as the internal standard; 0.2 ml lignocerane, 1 ml CH_2_Cl_2_, and 2 ml distilled water were added to each solution after first adjusting the solution to pH 3.4 with NaHCO_3_ (0.4 M). Subsequently, the organic phase was re-integrated into 0.4 ml CH_2_Cl_2_, homogenized, and the homogenate rotary evaporated at 45°C. Finally, the acetyl pyrimidine and LBSA technique and GC-MS were employed to, respectively, quantify lignin-derived monomers and total lignin content.

Constituent compounds in homogenized samples were separated using a TG-5MS (30 m ×0.25 mm ×0.25 μm) gas chromatography system (Thermo Fisher Scientific, Thermo, USA) with the following settings: front inlet temperature, 50°C; injection volume, 1.0 μl; helium flow rate, 1.2 ml/min; split ratio, 20:1; ionization, electron impact (EI); interface temperature, 28°C; ion source temperature, 280°C; and emission current, 70 eV. The oven program commenced at 50°C and rose to 310°C at a rate of 0.5°C/min, which was then held for 7 min. All analytical methods were performed as previously described (Shen et al., 2009).

### Quantitative Reverse-Transcription PCR

A first-strand cDNA synthesis kit was used to reverse transcribe 1 μg of total RNA (Tiangen, Co., Ltd., China). The obtained cDNA was then diluted 20 times and used as the template for real-time PCR. Six genes (i.e., *CCOAOMT, CAD, C4H, CCR, 4CL*, and *PAL*) were selected for expression validation by RT-qPCR, and specific primers were designed with Primer 5.0 (Supplementary Table S6). Gene expression was determined using a relative quantification approach with *PUB* as the housekeeping gene. Reaction mixtures (20 μl) consisted of DEPC-treated water and primer (0.8 μl), template cDNA (2 μl), and 1x SYBR Green real-time PCR Master Mix (10 μl). The thermocycler program, carried out on the Bio-Rad CFX96 Real-Time System (Bio-Rad CFX), consisted of 5 min at 95°C for denaturation and annealing, then extension at 55°C, repeated for 42 cycles and followed by a final 5 s at 95°C. Reaction products were evaluated using the Bio-Rad CFX Manager 2.0 software. Three biological and three technical replicates for each plant were used to ensure the reproducibility and reliability of the assay. Linear regression was used to calculate the efficiency (E) and correlation coefficient (*R*^2^) of each primer pair (Wang et al., 2019). Relative expression values were calculated using the 2^−ΔΔCT^ method, and degrees of differential expression were expressed in terms of fold change (Ding et al., 2018).

### Co-expression Analysis

The co-expression algorithm in the R software package was used to identify co-expression modules. When constructing modules, the power value threshold option was disabled. As the obtained power values ranged from 1 to 20, average and independence connection degrees of multiple modules were determined using the gradient technique. A degree of independence of 0.8 indicated a suitable power value. Once the power value threshold was established, modules were built using the WGCNA method. Genes relating to each module were also examined. To ensure high reliability of the findings, the minimum number of genes in a module was set at 30. The Cytoscape software was used to visualize co-expression networks (Rao et al., 2019).

### Statistical Analyses

Significant differences in experimental data were identified using Student's *t*-test with a criterion of *P* < 0.05, and are indicated by asterisks above bars in figures.

### Accession Numbers

The reference genome data utilized in this study were deposited in the NCBI Sequence Read Archive under accession number PRJNA529758. The RNA-seq data were deposited in NCBI under accession numbers SRS5227911-SRS5227946. The data supporting the results of this study are included in the article.

## Results

### Resistant and Susceptible Species of *Piper* Showed Significant Differences in Time-Course Gene Expression Profiles After Inoculation With *P. capsici*

**Figure 1:**
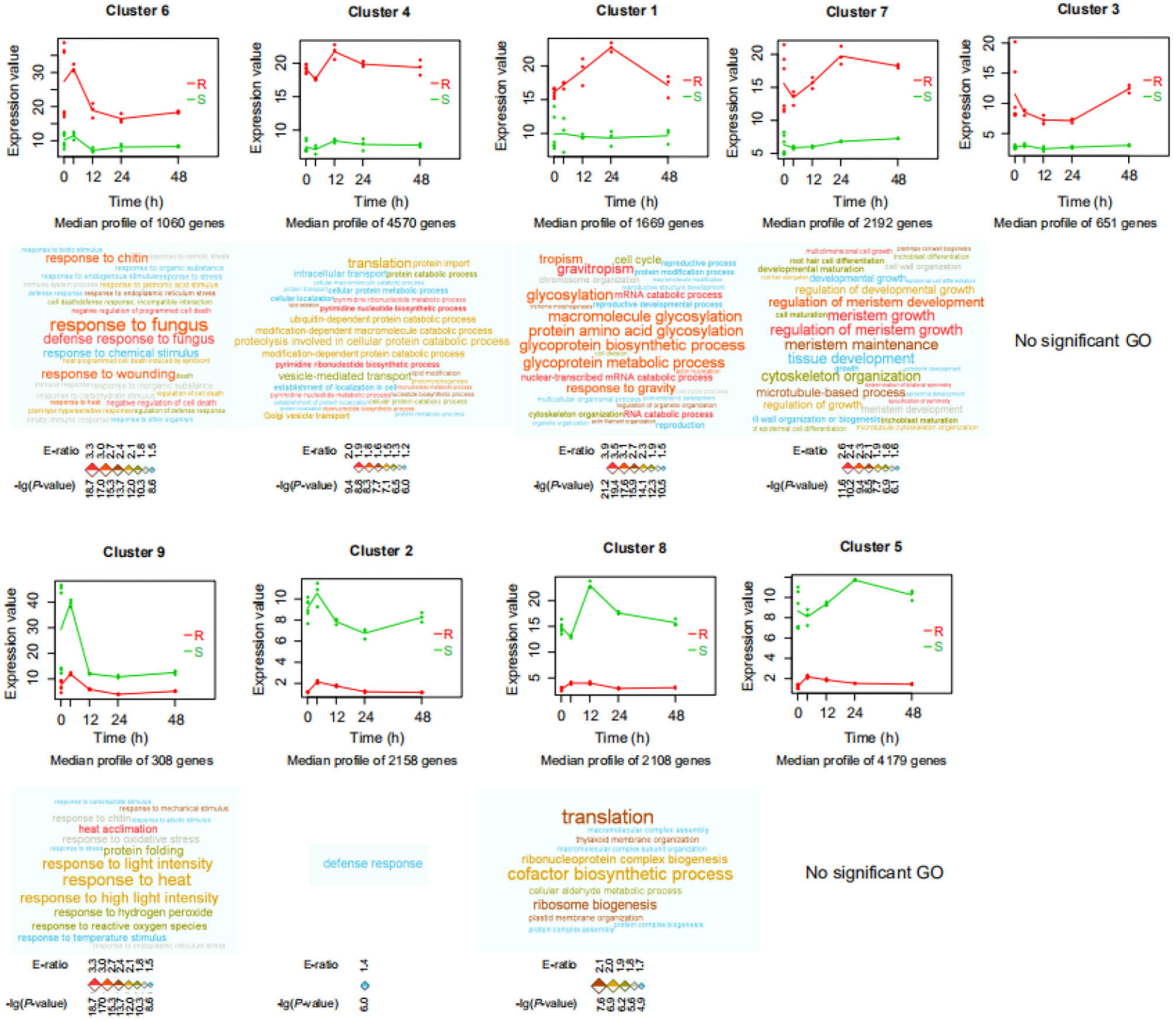
Significant differences in gene expression profiles between resistant (R; *P. flaviflorum*) and susceptible (S; *P. nigrum*) species of *Piper*. Expression profile clusters were identified by the *maSigPro* package from time-course RNA-Seq data of root samples at 0, 4, 12, 24, and 48 h post-*Phytophthora capsici* inoculation. x-axis: time; y-axis: gene expression. Red color indicates the resistant species, and green color indicates the susceptible species; lines represent the average of each time-group so as to visualize cluster-wide trends over time. Gene Ontology (GO) enrichment analysis was performed for the genes in each cluster; enriched biological process terms are visualized as word clouds below the cluster expression plots. Word clouds were generated by the WocEA software; font size and color denote the -log (*P*-value) and enrichment ratio (E-ratio), respectively.

**Figure 2:**
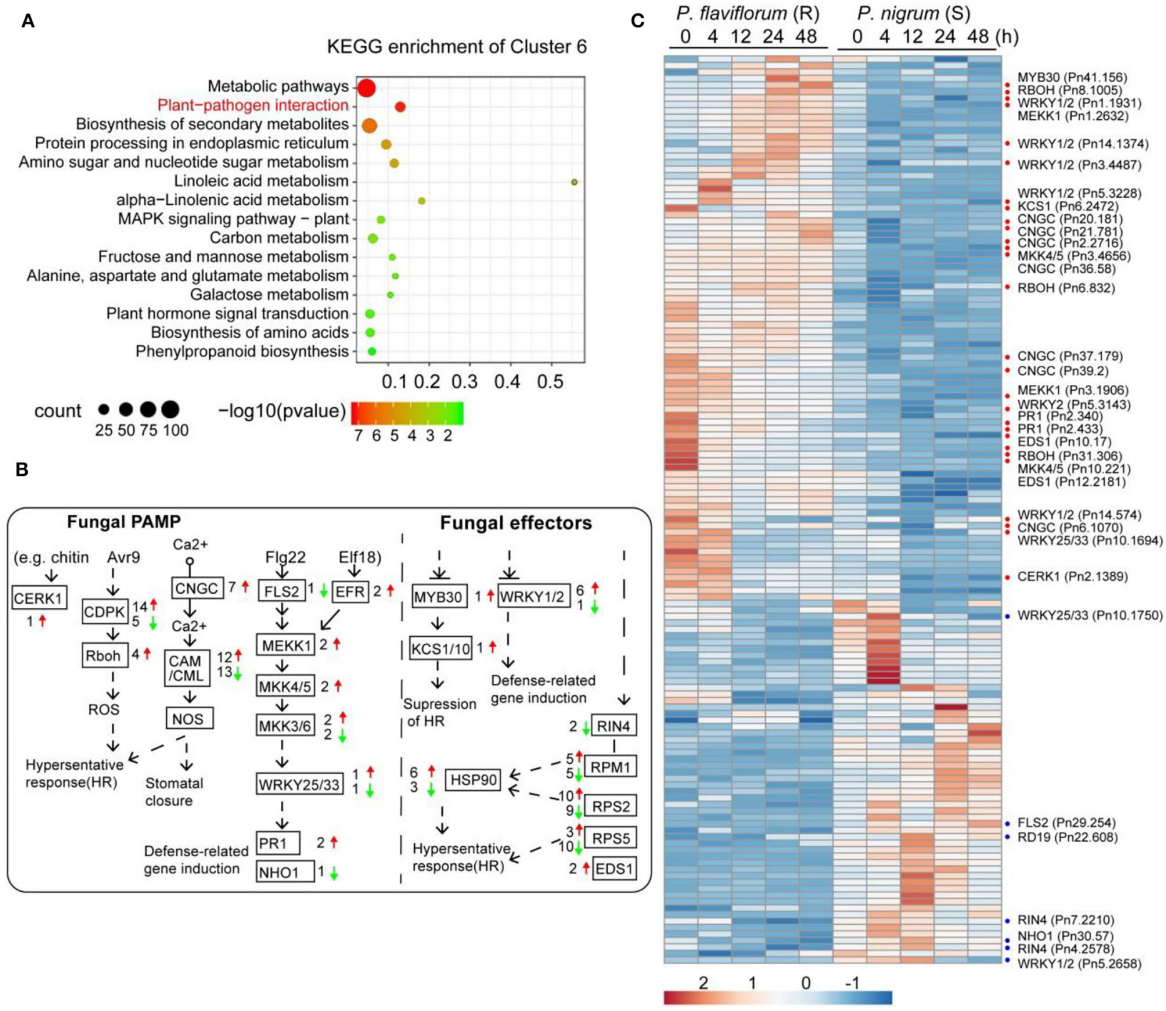
Profiles of differentially expressed genes in the plant-pathogen interaction pathway. **(A)** KEGG pathway enrichment analysis of genes in cluster 6 from Figure 1 revealed genes higher-expressed in resistant *Piper* (*P. flaviflorum*) to be significantly enriched in plant-pathogen interaction pathway members. **(B)** The KEGG plant-pathogen interaction pathway map, annotated with differentially expressed genes. Red arrows indicate higher and green arrows indicate lower expression in resistant *Piper*; numbers indicate the number of putative genes with differential expression. **(C)** Heatmap of the differentially expressed genes from panel b. For the detailed gene list, refer to Supplementary Table S2.

To determine differences in gene expression profiles between resistant (*P. flaviflorum*) and susceptible (*P. nigrum*) species of *Piper* infected with *P. capsici*, we conducted mRNA-seq at five different time points (0, 4, 12, 24, and 48 h) after inoculation with *P. capsici* (Supplementary Table S1). From the resulting data, clusters of genes with similar expression patterns were identified using the *maSigPro* package. In total, 18,895 genes were found to cluster into nine groups (Figure 1; Supplementary Table S2). Genes in clusters 6, 4, 1, 7, and 3 had relatively higher expression in the resistant *P. flaviflorum* than in the susceptible *P. nigrum*, whereas for the other four clusters, the situation was reversed. GO enrichment analysis revealed that the genes in cluster 6 are significantly enriched in terms relating to plant defense responses, including “response to fungus,” “response to chitin,” and “response to wounding” (Figure 1). Similarly, KEGG analysis of cluster 6 identified significant enrichment of 15 categories including “plant-pathogen interaction,” “plant hormone signal transduction,” and “phenylpropanoid biosynthesis” (Figure 2A).

To develop a more detailed understanding of differentially expressed genes and their role in *Phytophthora* infection, we first focused on genes involved in plant-pathogen interactions, including pathogen-associated molecular pattern (PAMP)-triggered immunity (PTI) and effector-triggered immunity (ETI), which are two bases of plant immunity defense. In total, 140 genes involved in these pathways were found to have differential expression in the resistant *P. flaviflorum* compared with the susceptible *P. nigrum* (Figures 2B,C; Supplementary Table S3); these included *CNGC*s, *CDPK, RBOH, CaM/CML, MEKK1, MKK4/5, Pit1, RPM1, RPS2, PBS1, EDS1, HSP90, WRYK33*, and *PR1*.

### Resistance of *P. flaviflorum* to *P. capsici* Is Conferred by Modulation of Phytohormone Biosynthesis, Transduction, and Signaling

**Figure 3:**
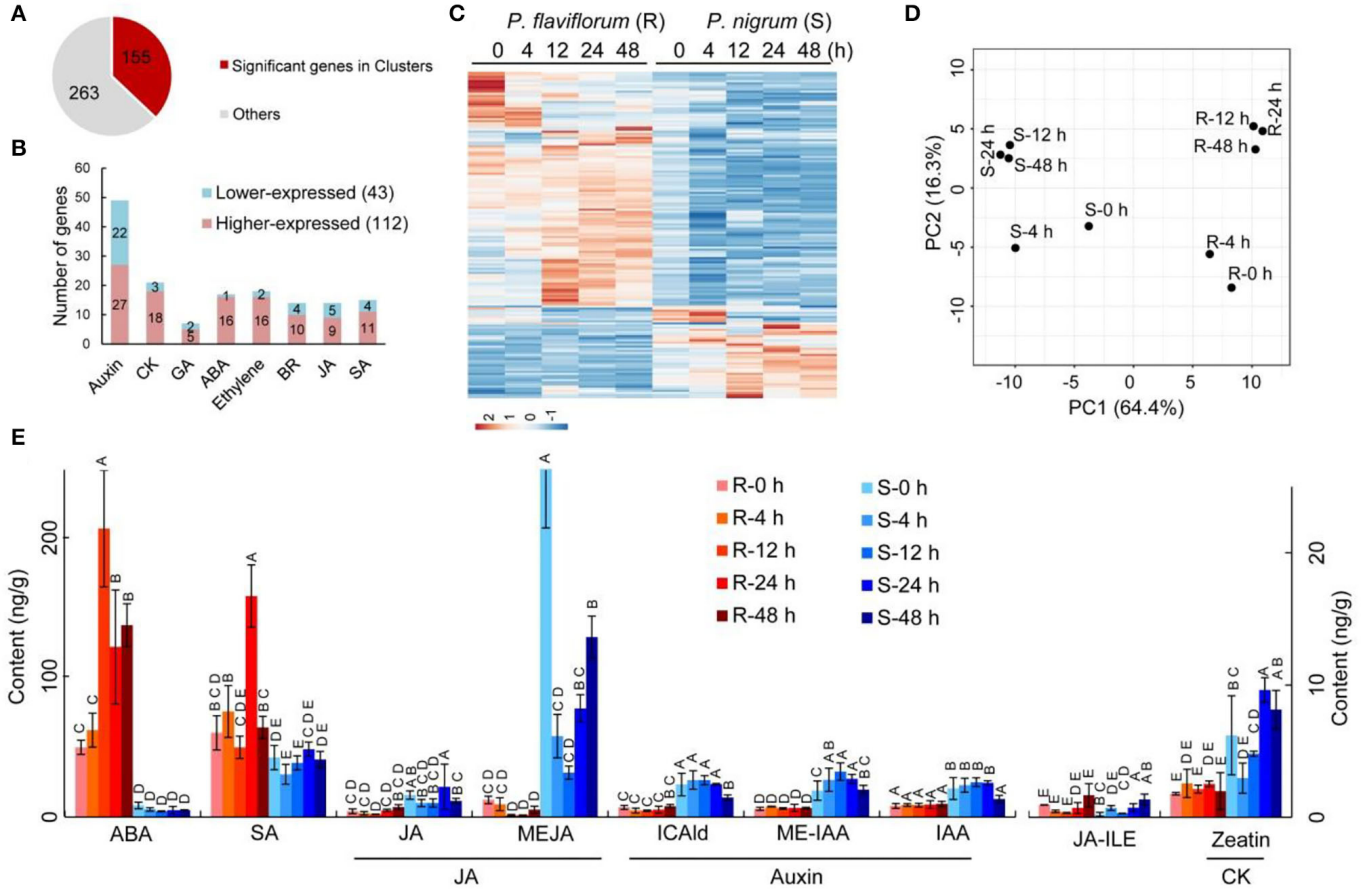
Time-course-dependent accumulation of ABA and SA and patterns of differential expression for genes involved in phytohormone signaling and response in resistant *Piper* (R; *P. flaviflorum*) upon *P. capsici* inoculation. **(A)** Number and proportion of differentially expressed genes relative to all putative genes involved in phytohormone signaling. **(B)** Pathway distribution of the 155 differentially expressed genes involved in plant hormone signal transduction. Red color indicates higher and blue color indicates lower relative expression in resistant *Piper* compared with susceptible *Piper*. **(C)** Heatmap of genes from **(B)**. **(D)** Principal component analysis plots of significant cluster genes involved in plant hormone signal transduction, based on RNA-seq data. For clarity, each dot represents the average expression from three replicates. **(E)** Time-course LC-MS/MS evaluating the plant hormone content of root samples at 0, 4, 12, 24, and 48 hpi, including abscisic acid (ABA), salicylic acid (SA), jasmonates (JA), auxins, and cytokinin (CK).

We further examined genes involved in phytohormone signaling and response. In total, 418 genes were annotated with the KEGG pathway “plant hormone signal transduction,” of which 155 genes clustered into the nine significant expression groups (Figure 1) while the other 263 genes were similarly expressed without cluster information (Figure 3A). Among the 155 significant genes, 43 genes were lower expressed and 112 genes were higher expressed in *P. flaviflorum* infected with *P. capsici*. These genes represented a number of phytohormone signaling and response pathways, including auxin, cytokinins (CKs), gibberellic acid (GA), abscisic acid (ABA), ethylene, brassinosteroids (BRs), jasmonic acid (JA), and salicylic acid (SA) (Figures 3B,C; Supplementary Table S5).

Specifically, three genes in the ABA signaling pathway were identified as having significantly different expressions, namely, *PP2C, PYL*, and *ABF*. A group of genes encoding proteins involved in jasmonate signaling exhibited higher expression in resistant than in susceptible plants; these included *COI1, JAR1*, and *JAZ3* (Supplementary Table S5). Differentially expressed genes involved in SA biosynthesis included *ABF, GIA, DPBF, ABI, SAG*, and *PYL*, along with eight genes encoding NPR proteins, which are key regulators of the SA-mediated defense response; these eight genes were upregulated during the defense response in *Piper* species, with *NPR1* and *NPR3* showing particularly high activity in resistant plants at 24 h post-inoculation (hpi), but only minor increase in susceptible plants. Collectively, these findings indicate that the response of *Piper* plants to *P. capsici* infection involves multiple phytohormone signaling pathways and responses.

Subsequently, principal component analysis (PCA) was performed to determine the expression patterns of significant genes at various time points. This analysis revealed that the two species of *Piper* are significantly separated from each other on PC1 (64.4%), and the time points of 12, 24, and 48 h are clustered together within each species on PC2 (16.3%), indicating that after *P. capsici* infection, the major phytohormone response is established by 12th hour and largely completed by 48th hour (Figure 3D).

To further understand the cooperation between phytohormones in *Piper* plants following *P. capsici* infection, we measured the levels of ABA, SA, JA (including JA, JA-ILE, and MEJA), auxin (including ICAld, ME-IAA, and IAA), and cytokinin (including zeatin). The resistant *P. flaviflorum* exhibited a background (i.e., 0 hpi) reservoir of ABA that was ~6-fold higher than that of the susceptible *P. nigrum*, along with 4.1-fold increased accumulation of ABA at 12 hpi compared with its 0 hpi time point, whereas no significant change of ABA level was observed in *P. nigrum* after infection. Meanwhile, the two species had equivalent background contents of SA, but *P. flaviflorum* showed 2.6-fold increased accumulation at 24 hpi, while *Piper nigrum* had no significant increase of SA accumulation after infection. In contrast, JA was implicated in the *P. nigrum*-*P. capsici* interaction, with the susceptible species exhibiting increased MEJA and JA-Ile at 48 hpi. The other two phytohormones, namely, auxin and cytokinin, were found to have significantly higher background reservoirs in susceptible *P. nigrum*, but no significant postinfection changes in their levels (Figure 3E).

### Resistance of *P. flaviflorum* to *P. capsici* Is Conferred by Modulation of Phenylpropanoid and Lignin Biosynthesis

**Figure 4:**
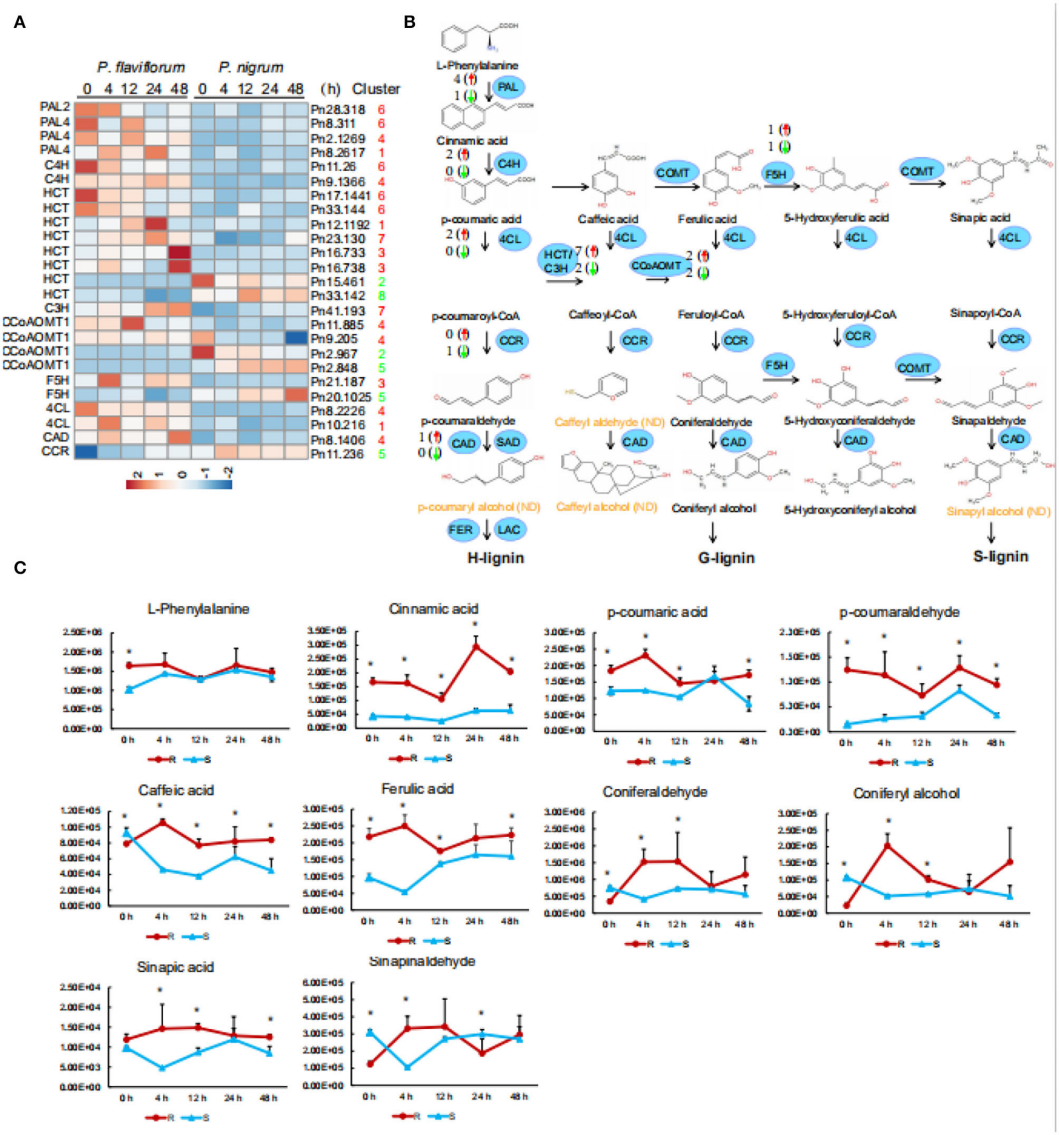
Enriched accumulation of lignin precursors in resistant *Piper* (*P. flaviflorum*). **(A)** Heatmap of all differentially expressed genes in the lignin biosynthesis pathway. **(B)** Diagram of the lignin biosynthesis pathway, annotated with differentially expressed genes. Red arrows indicate higher and green arrows lower expression in resistant *Piper*; numbers indicate the number of putative genes with differential expression. ND, not detected. **(C)** Time-course-dependent accumulation of lignin precursors in the LC-MS/MS data of root samples at 0, 4, 12, 24, and 48 hpi. R: resistant *Piper* (*P. flaviflorum*); S: susceptible *Piper* (*P. nigrum*).

To investigate the functions of phenylpropanoids and lignins in pepper infected with *P. capsici*, we examined the 25 genes involved in phenylpropanoid biosynthesis that were members of expression clusters. These included four *PAL* genes, two encoding 4-coumarate-CoA ligase (*4CL*), eight encoding hydroxy cinnamoyl transferase (*HCT*), one *C3H* gene, two *F5H* genes, four encoding caffeoyl-CoA O-methyltransferase (CCoAOMT), one encoding cinnamyl alcohol dehydrogenase (CAD), and one encoding cinnamoyl CoA reductase (CCR). After *P. capsici* infection, these genes were generally more highly expressed in *P. flaviflorum* than in *P. nigrum*, except for two *HCT* genes, two *CCoAOMT* genes, one *F5H gene*, and *CCR* (Figures 4A,B). Furthermore, qPCR performed on samples from inoculated plants showed that the expression of *PAL* in plant roots was stimulated after 4 h and continuously increased thereafter. In addition, resistant plants showed higher *PAL* activity after 24 h than did susceptible plants (Supplementary Figure S2). Generally, these results illustrate the activation of the phenylpropanoid pathway as part of the defense response of *Piper* species to *P. capsici* infection.

We additionally detected by LC-MS/MS ten lignin precursors (i.e., L-phenylalanine, cinnamic acid, p-cinnamic acid, p-coumaraldehyde, caffeic acid, ferulic acid, coniferaldehyde, coniferal alcohol, sinapic acid, and sinapinaldehyde) in the roots of *P. flaviflorum* and *P. nigrum* after *P. capsici* infection (Figure 4C). Compared with the susceptible *P. nigrum*, the resistant *P. flaviflorum* exhibited higher levels of L-phenylalanine, cinnamic acid, p-cinnamic acid, p-coumaraldehyde, ferulic acid, and sinapic acid at 4, 12, 24, and 48 hpi, although p-coumaric acid, coniferyl alcohol, and sinapinaldehyde were lower at 24 h (Figure 4C). It is interesting to note that most precursors in lignin metabolism remained at higher levels in *P. flaviflorum* than in *P. nigrum*.

We further applied GC/MS to analyze lignin monomer compositions in inoculated stems of the two *Piper* species. Analysis of thioacidolysis products revealed the stems of these plants to contain higher proportions of guaiacyl (G) and syringyl (S) units and a lower proportion of p-hydroxyphenyl (H) units, with H units only accounting for 0.19–0.37 mg/g. Notably, inoculated resistant plants exhibited higher levels of H units than did susceptible plants. For inoculated resistant plants, G units comprised 28.70 mg/g at 21 dpi; however, inoculated susceptible plants were dead. The G/S ratios increased compared with the control in resistant species of *Piper*, resistant black pepper inoculation exhibited increased G/S ratios going from 2.81 to 6.87 mg/g at 21 dpi. In contrast, the G/S ratio of susceptible black pepper only increased from 1.85 to 1.89 mg/g after *P. capsici* inoculation (Table 1).

**Table 1:** Stem lignin content and monomer composition in the two species of *Piper* after inoculation with *P. capsici*.

	**Time after inoculation (day)**	**Lignin content (g/100 g)**	**Monomer content (mg/g)**			
			**H**	**G**	**S**	**G/S**
*Piper nigrum*	Control	15.43 ± 0.22a	0.24 ± 0.003b	12.53 ± 0.15c	6.76 ± 0.08e	1.85
	7	21.17 ± 0.47b	0.19 ± 0.0013a	8.54 ± 0.09a	4.50 ± 0.02d	1.89
	21	27.99 ± 0.86c	–	–	–	–
*Piper flaviflorum*	Control	21.31 ± 0.19b	0.37 ± 0.003d	10.22 ± 0.26b	3.63 ± 0.02a	2.81
	7	28.67 ± 0.03c	0.27 ± 0.007c	21.52 ± 0.05d	3.94 ± 0.03b	5.46
	21	34.75 ± 0.09d	0.24 ± 0.005b	28.70 ± 0.21e	4.18 ± 0.04c	6.87

*Values within a column followed by different letters indicate significant difference (P < 0.05)*.

*G, guaiacyl; S, syringyl; H, p-hydroxyphenyl; –, not detected*.

### Co-expression Networks of Lignin Biosynthesis Genes in *P. flaviflorum* and *P. nigrum*

**Figure 5:**
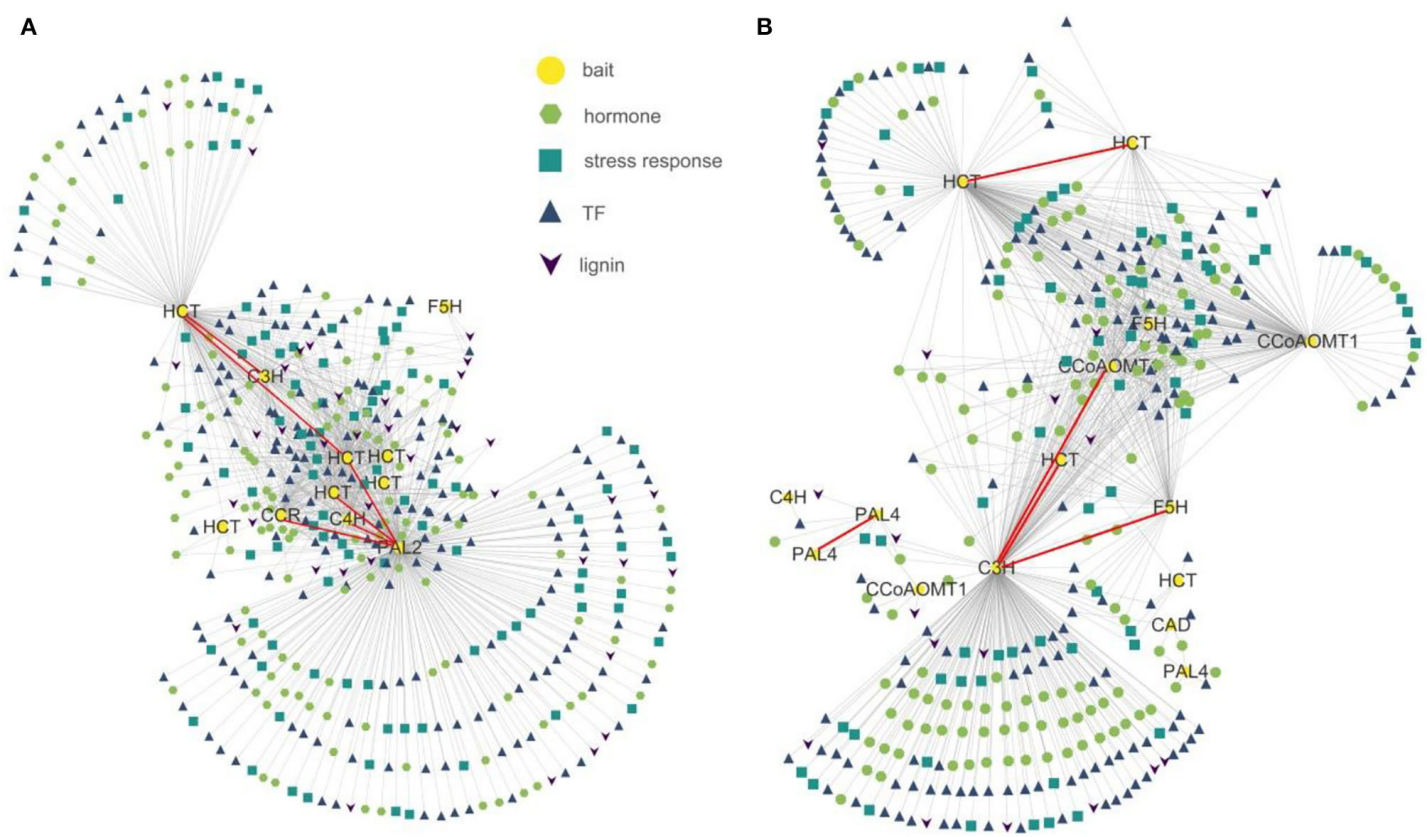
Co-expression networks showing the relationships of lignin biosynthesis genes having differential expression in resistant (*P. flaviflorum*) and susceptible (*P. nigrum*) species of *Piper* with transcription factor, phytohormone response, and stress response genes. **(A)** Network for *P. flaviflorum*. **(B)** Network for *P. nigrum*.

To detect co-expression profiles of genes associated with lignin biosynthesis, we applied a modified analysis method based on QUBIC, a previously reported bi-clustering algorithm (Li et al., 2009; Zhang et al., 2017). We utilized as bait genes 25 significant lignin biosynthesis genes from clusters (Supplementary Table S4) to identify co-expressed TF, phytohormone signaling, stress response, and lignin biosynthesis genes also differentially expressed in the RNA-seq data of *P. nigrum* and *P. flaviflorum* root samples at 0, 4, 12, 24, and 48 hpi and then compared co-expression results between the two species. In *P. nigrum*, this analysis identified 152 transcription factors, 132 phytohormones, 70 stress responses, and 19 lignin biosynthesis genes as co-expressed with 15 of the bait genes, while for *P. flaviflorum*, it yielded 182 transcription factors, 130 phytohormones, 98 stress responses, and 37 lignin biosynthesis genes that were co-expressed with 11 bait genes (Figure 5). In both species, the expression of the various target gene types correlated significantly with the expression of *PAL, HCT, C4H, C3H, F5H*, and *CCR*, but not with *CCoAOMT, 4CL*, or *CAD*; this suggests that *P. nigrum* and *P. flaviflorum* have evolved a conserved mechanism of disease resistance that recruits homologous members of certain TF families, phytohormone signaling pathways, stress responses, and the lignin biosynthesis pathway. However, the total number of co-expressed lignin biosynthesis genes was higher in *P. flaviflorum* than in *P. nigrum*, indicating more complicated interactions between hormone signaling and the action of transcription factors in the resistant species when fighting off infection.

We characterized the phenotypes of resistant and susceptible species following inoculation (Supplementary Figures S1A–C). After infection by *P. capsici*, the root system, the base of the main vine, the branches, the leaves, the flowers, and the fruits of black pepper plants were all damaged (Supplementary Figure S1A). However, leaf browning and stem browning were only observed in the susceptible species; no external lesions were observed in resistant plants (Supplementary Figures S1B,C). In addition, tracing the growth of resistant and susceptible *Piper* plants for 50 days revealed a significantly higher increase in dry weight of roots for the susceptible *P. nigrum* as compared with the resistant *P. flaviflorum* (Supplementary Figure S1D), indicating a possible trade-off between defense and growth.

**Figure 6:**
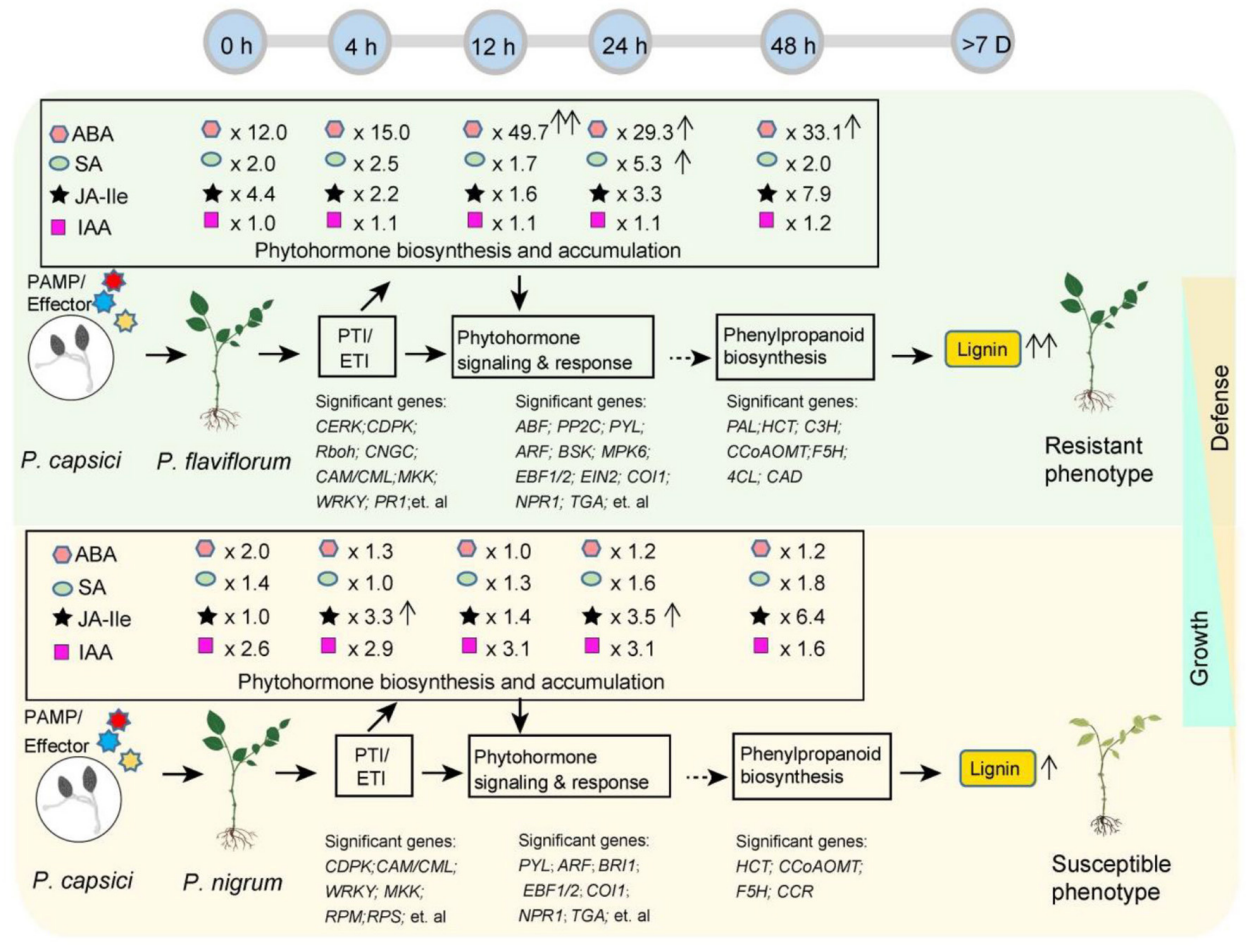
Schematic summarizing the different transcriptome and metabolome profiles in resistant *P. flaviflorum* and susceptible *P. nigrum* upon *P. capsici* infection. Significant differences were observed in the expression of PTI and EIT pathway genes, endogenous phytohormone levels, phytohormone signaling and response, and also in the downstream phenylpropanoid biosynthesis pathway and lignin levels, leading to different postinfection phenotypes in resistant vs. susceptible *Piper*. Time-course ABA, SA, JA-Ile, and IAA levels were normalized and are presented as the relative content in resistant *Piper* compared with susceptible *Piper*; arrows after relative ratios indicate a significant increase (*P* < 0.05) in phytohormone levels at the indicated time point relative to 0 h for that species.

Figure 6 depicts a schematic diagram summarizing the different transcriptome and metabolome profiles in resistant *P. flaviflorum* and susceptible *P. nigrum* following infection with *P. capsici*. The two species showed significantly different expression patterns for genes involved in the PTI and ETI pathways, endogenous phytohormone accumulation, phytohormone signaling and response, and also the downstream phenylpropanoid biosynthesis pathway; they also exhibited differential lignin accumulation levels. Representative significant genes in plant-pathogen interaction (PTI/ETI), phytohormone signaling and response, and phenylpropanoid biosynthesis pathway were listed for both *Piper* species. Importantly, *P. flaviflorum* was observed to have a higher background endogenous ABA reservoir, increased accumulation of ABA at 12 hpi, and increased accumulation of SA at 24 hpi. These accumulations of ABA and SA, but not JA, appear to play important roles in resistant *P. flaviflorum* after *P. capsici* infection. In contrast, the susceptible *Piper* exhibited time-course-dependent accumulation of JA, but not ABA or SA, along with a higher background endogenous auxin (IAA) reservoir and a higher growth rate consistent with that reservoir (Supplementary Figure S1). The differences in phytohormone accumulations and gene expression profiles in biologically important pathways lead to different accumulations of lignin content and thus the different defense and growth phenotypes of these two species (Figure 6).

## Discussion

### Differences in Plant-Pathogen Interaction Between Resistant and Susceptible Species of *Piper*

Using transcriptome, metabolome, and co-expression network analysis, this study revealed an array of differences between *Phytophthora-*resistant and *Phytophthora*-susceptible species of *Piper*. In particular, significant differences in the immune regulation network were observed, suggesting the resistance of *P. flaviflorum* might be linked through the specific expression of immune genes to the activation of related downstream genes and hence an efficient immune response process. We further illustrated the differences in immune regulation between the two *Piper* species and described the immune regulation process of the resistant pepper. Analyses were conducted at five time points of 0, 4, 12, 24, and 48 h post-inoculation (hpi), selected to correspond with critical phases of pathogen infection and for isolating the early pathogen-responsive genes. The period within the first 12 hpi usually corresponds to pathogen spore germination and penetration of epidermal cells (Fradin and Thomma, 2006). At time zero, *P. flaviflorum* exhibited higher average expression of gene clusters significantly enriched for KEGG pathways such as plant-pathogen interaction, plant hormone signal transduction, or phenylpropanoid biosynthesis, for example, cluster 6 (Figure 2A); this finding suggests these pathways to have higher background expression in the resistant species. The early phases of pathogen infection often involve complex exchange, perception, and transduction of signals (Kim et al., 2013). The interplay of signals that enables the plant to fine-tune defensive responses was described in detail by elucidating the responses of associated genes.

This study identified significantly different expressions in *P. flaviflorum* for many genes associated with the ETI (pathogen effector-induced immunity) and PTI (pathogen-associated molecular pattern-induced immunity) aspects of plant immunity, including *CERK1, CDPK, FLS2, CaMCML, MEKK1, MKK4/5, WRYK33, PR1, EFR*. Of particular note is *CERKl*, which is genetically and biochemically defined as a receptor for chitin and may activate PTI by activating downstream *CDPK*, inhibiting the proliferation of pathogenic bacteria (Zeng et al., 2015). Another important PTI pathway is represented by *FLS2*, which is related to the recognition of foreign pathogen invasion and activation of downstream resistance genes (Zipfel et al., 2004). In *P. flaviflorum, FLS2* may initiate the amplification of *MAPK* and subsequently launch PTI through activating transcription of disease-resistance genes such as *WRKY* and *ERF* (Boller and Felix, 2009).

Furthermore, we found that the RLK protein kinase subfamily is highly expressed in *P. flaviflorum*. This represents another critical factor in the defense system against *P. capsici* due to members of this family having roles in toggling the activity of downstream target proteins through phosphorylation or dephosphorylation and transmitting extracellular signals to other cells. *RLK* genes are known to be important in plant development, disease resistance, and phytohormone perception and have been cloned from Arabidopsis, rice, wheat, and other plants (Gao et al., 2013). In addition to the expression of the receptor kinase being significantly higher in *P. flaviflorum*, specific genes upregulated during the early stage in *P. flaviflorum* had different expression patterns in *P. nigrum*. The relative lack of receptors in *P. nigrum* and lower expression of early-stage genes might prevent the timely recognition of fungal infection signals, thus leading to the different responses in these two species. Similar scenarios have been reported in previous studies (Feys et al., 2001); for example, in tomato, *EDS1* is an important regulatory factor in pathogen defense (viruses, bacteria, fungi, etc.), an essential regulator of the resistance genes *VE1* and *VE2*, and an effective regulator of resistance genes in the TIR-NBS-LRR family.

Our results also identified that some TFs are likely to play critical regulatory roles in the resistance of *Piper* species, particularly members of the plant-specific WRKY family. This is one of the most diverse families of transcriptional regulators in plants, and its members are essential components of signaling webs that control a variety of plant activities (Rushton et al., 2010); they comprise key plant stress response genes and are involved in multiple immune regulation processes, including both ETI and PTI. WRKY TFs have been reported to promote secondary metabolite synthesis, enhance plant salt tolerance, participate in response to fungus infection (Loo et al., 2020), and contribute to bacterial resistance. For example, overexpression of *OsWRKYL3* in rice can increase resistance to bacterial blight and rice blast, and it contributes to the disease resistance response mediated by the major resistance factor *Xa3/Xa26* (Qiu et al., 2007). Meanwhile, the expression of *OsWRKY31* has been shown to be induced by *Magnaporthe grisea*, which could enhance plant resistance to this pathogen, affect root growth, and participate in the auxin response (Zhang et al., 2008). Findings from this study indicate WRKY to be involved in PTI for both investigated species of *Piper*, interacting with *P. capsici* and the network regulating lignin synthesis.

### Differences in Phytohormone Biosynthesis and Responses Between Resistant and Susceptible Species of *Piper*

Specific accumulation curves of several key hormones were observed in resistant *Pipe*r, indicating that phytohormone synthesis and responses play crucial roles in the resistance of *Piper* species to *Phytophthora*. A previous report (Cheng et al., 2016) identified ABA, SA, and JA as plant “stress hormones” and as significant contributors to plant responses to abiotic stresses. In this study, significant differences in ABA content were evident in the context of *P. capsici* infection. Specifically, the resistant *P. flaviflorum* showed a high background endogenous ABA reservoir and time-course-dependent accumulation at 12 hpi, which preceded the increase in SA accumulation at 24 hpi, indicating ABA to have a crucial early role in the infection process for the resistant species. Taken together, the phytohormone assay results indicate that resistant and susceptible *Piper* species have substantial differences in phytohormone background reservoir levels and in their accumulation after *P. capsici* infection. Moreover, ABA and SA may play major roles in the *P. flaviflorum*–*P. capsici* interaction. The resistance mechanism of ABA has been revealed to some extent, and examples of ABA-mediated resistance have been reported (Cheng et al., 2016). Previous studies have shown ABA to not only significantly increase the expression of abiotic stress resistance genes but also increase the expression of most resistance-related genes, including *AP2/EREBPs*, NBS-LRRs, *PAL*, serine/threonine-protein kinases, *PPO*, chitinase, and peroxidase (*POD*). In addition, it also upregulates the genes involved in the jasmonic acid, salicylic acid, and ethylene signaling pathways (Song et al., 2011). Recently, it was found that ABA can activate Ser/Thr protein kinase AAPK expression and hence regulate the signal transduction pathway of stomatal closure induced by S-type anion channels in guard cells (Li et al., 2000). In *OsERF922* overexpression lines, which exhibit significantly increased resistance to *Magnaporthe oryzae*, the expression of ABA synthesis genes *NCED3* and *NCED4* is significantly increased, as is the expression of defense genes (Liu et al., 2012). Therefore, one of the ways that ABA induces plant resistance to pathogens may be through regulating mRNA expression and related genes and activating plant defense mechanisms, thus causing a series of physiological and biochemical changes.

The SA is the primary mediator of plant resistance to pathogens. During invasion by a pathogen, plants accumulate a large amount of SA and activate the SA-mediated signaling pathway of disease resistance (Dong, 2001). Subsequently, *NPR1* is depolymerized into monomers and moved into the nucleus, where it combines with TGA family transcription factors (Yang et al., 2015). *NPR1* also activates the expression of downstream WRKY TFs and glutathione protein genes, which can directly or indirectly inhibit the expression of key genes of the JA signaling pathway (*COI, JAZ, MYC*) and so inhibit that pathway (Van der Does et al., 2017). The results of this study indicate that SA plays an important role in the resistance of *P. flaviflorum* to the pathogen *P. capsici*, as SA content peaked at 24 hpi. Moreover, the set of significantly differentially expressed genes included a number involved in SA signal transduction, such as *NPR1, NPR3*, and *PAD*. Further analysis of the expression patterns of these genes in both species of *Piper* after infection revealed that the speed and degree of their induction at 12 hpi are significantly higher in *P. flaviflorum* than in *P. nigrum*.

Protein-protein interaction network analysis further confirmed the difference between susceptible and resistant species of *Piper* in the network of interactions invoked in response to *P. capsici*. Hub genes in the susceptible network included those associated with JA signaling, suggesting that JA-mediated induced resistance (IR) might contribute to the molecular mechanisms deployed by susceptible *Piper* species against *P. capsici*, especially in the early phase of their interaction. In contrast, the systemic acquired resistance (SAR) associated with the SA or ABA pathways might contribute to mechanisms in play against *P. capsici* in resistant species of *Piper*. Our results concerning plant hormone contents also supported this conclusion.

### G-Lignin in *Piper* Species Resistance to *P. capsici*

Strategies used by plants to efficiently limit pathogen transmission in the vascular system include altering lignin monomer composition and cross-linking in order to strengthen the cell wall (Gayoso et al., 2010). Lignins are synthesized through the oxidative coupling of any of three monolignols, namely, syringyl (S), p-hydroxyphenyl (H), and guaiacyl (G) (Ding et al., 2018), with plant species and tissue type influencing the relative proportions of these three major components in the cell wall. In addition, most secondary metabolites in the plant phenylpropanoid pathway are considered to be plant defense compounds; lignin, which is downstream of the phenylpropanoid pathway, might likewise contribute to plant disease resistance.

Thioacidolysis was applied to investigate the lignification in susceptible and resistant species of *Piper* following exposure to *P. capsici*. Examination of thioacidolysis products indicated that black pepper stem lignin has a larger proportion of S and G units and a smaller proportion of H units, which is consistent with the notion that dicotyledon lignin is mostly comprised of S and G units. At both 7 and 21 days following inoculation, the stems of the resistant *Piper* species had a higher G/S ratio than those of the susceptible *Piper* species. In general, the resistant species exhibited higher levels of all three unit types, with G lignin in particular being thought to have a key part in its defense response. These findings are consistent with the analysis of root lignin by LC-MS/MS; namely, at post-inoculation time points, the roots of *P. flaviflorum* exhibited greater content than *P. nigrum* for all ten lignin precursors except for p-coumaric acid, coniferyl alcohol, and sinapinaldehyde at 24 hpi. We speculate that the increase of G-type lignin following infection would make for more compact cross-linking between lignin monomers. According to previous research, the lignin pathway is activated in incompatible path systems, since reinforcement and lignification of cell walls are key processes in plant responses to fungal infection (Naoumkina et al., 2010). For example, lignin synthesis and deposition were investigated in cotton hypocotyls exposed to an elicitor of *Verticilium dahliae* and were revealed to play an important part in plant response to that pathogen. In particular, lignin is thought to provide a physical barrier, making the cell wall more resistant to mechanical pressure throughout fungal penetration (Smit and Dubery, 1997). Consistent with the results of this study, a study in two varieties of flax mustard with differing *Sclerotinia sclerotiorum* resistance found the G/S ratio of lignin monomers to be greater in resistant plants relative to susceptible plants (Eynck et al., 2012). Hence, lignin content and its monomer ratio are closely related to crop disease and stress resistance and thus can be used as an important index for resistance evaluation. We also found that after inoculation with *P. capsici*, the rate of lignin accumulation in *P. flaviflorum* was significantly higher than that in *Piper nigrum*, mainly mediated by the synthesis and polymerization of G-type lignin monomers; this indicates that infection also triggered the polymerization of lignified monomers into lignin.

In addition to their roles as signaling molecules, phenylpropanoid products may also contribute significantly to plant defense responses through their phytoalexin functions (Ding et al., 2015). In this study, flavonoid accumulation in the roots of the two *Piper* species indicates the possibility of flavonoids serving as signaling molecules. Further transcriptional study of genes related to the phenylpropanoid pathway may help extend our comprehension of the molecular mechanisms underlying the differential defense responses of these two *Piper* species to *P. capsici*.

## Data Availability Statement

The datasets presented in this study can be found in online repositories. The names of the repository/repositories and accession number(s) can be found below: https://www.ncbi.nlm.nih.gov/bioproject/PRJNA529758.

## Author Contributions

C-yH and Q-hW conceived the study. RF, X-yT, Z-qX, L-sH, and B-dW participated in the preparation of experimental materials. C-yH, RF, and B-dW participated in RNA extraction. X-yT analyzed the data. L-sH performed qPCR. RF, X-yT, and SS wrote the manuscript. All authors read and approved the final manuscript.

## Funding

Financial support was provided by the Introduction of International Advanced Agricultural Science and Technology of the Ministry of Agriculture (2010-G2), the China Agriculture Research System (CARS-11), the specific research fund of the Innovation Platform for Academicians of Hainan Province (YSPTZX202154), the Natural Science Foundation of Hainan Province of China (321RC652), and the Natural Science Foundation of China (No. 31601626) and is gratefully acknowledged.

## Conflict of Interest

The authors declare that the research was conducted in the absence of any commercial or financial relationships that could be construed as a potential conflict of interest.

## Publisher's Note

All claims expressed in this article are solely those of the authors and do not necessarily represent those of their affiliated organizations, or those of the publisher, the editors and the reviewers. Any product that may be evaluated in this article, or claim that may be made by its manufacturer, is not guaranteed or endorsed by the publisher.

## Supplementary Material

Supplementary Figure S1 Phenotypes of resistant and susceptible species following inoculation with *P. capsici* (**A**: Plants in the field; **B**: Stems; **C**: Leaves; **D**: Dry weight of roots).

Supplementary Figure S2 Detailed expression profiles of major lignin biosynthetic pathway genes in the roots of black pepper. Error bars denote the standard error from three biological replications. x-axis labels indicate treatment group and time point: CK, control; no prefix, inoculated with *P. capsici*; numbers, hours post-infection. The y-axis represents gene expression level relative to that of ubiquitin. Each column represents the mean value plus standard deviation from three biological replicates.

Supplementary Table S1 Metadata from mRNA-seq.

Supplementary Table S2 Details of all differentially expressed profiles (clusters).

Supplementary Table S3 Details of differentially expressed profiles (clusters) including genes involved in plant-pathogen interaction.

Supplementary Table S4 Details of differentially expressed profiles (clusters) including genes involved in plant hormone signal transduction.

Supplementary Table S5 Details of differentially expressed profiles (clusters) including genes involved in the lignin synthetic pathway.

Supplementary Table S6 Primers used for qPCR.
