# Supplementary material for: Comparative Transcriptome and Metabolome Analysis of Resistant and Susceptible Piper Species Upon Infection by the Oomycete Phytophthora Capsici
Source: Front Plant Sci. 2022 Jun 23;13:864927. doi: 10.3389/fpls.2022.864927 (PMC9278165; doi:10.3389/fpls.2022.864927)
Supplement: Supplementary Table S1 — Metadata from mRNA-seq. [file Table_1.DOCX]

Table S1 Metadata from mRNA-seq

| Sample | Stage | Biological replicates | Raw reads | Clean reads | Total records | Mapped reads | Unique reads |
| --- | --- | --- | --- | --- | --- | --- | --- |
| ***P. nigrum*** | Control | 1 | 28385176 | 28383083 | 66003328 | 49482462 (74.97%) | 35501986 (53.79%) |
|  |  | 2 | 24976555 | 24974507 | 56886458 | 47996267 (84.37%) | 36838244 (64.76%) |
|  |  | 3 | 27056922 | 27055273 | 61952126 | 50431984 (81.4%) | 38028071 (61.38%) |
|  | 0 h | 1 | 19965484 | 19964332 | 45300056 | 40932279 (90.36%) | 32135651 (70.94%) |
|  |  | 2 | 23144763 | 23142847 | 53257960 | 38048755 (71.44%) | 27453998 (51.55%) |
|  |  | 3 | 26397072 | 26395582 | 59079714 | 45405058 (76.85%) | 35242668 (59.65%) |
|  | 4 h | 1 | 30376168 | 30734379 | 72905166 | 65759829 (90.2%) | 48069805 (65.93%) |
|  |  | 2 | 24166942 | 24165230 | 55181078 | 50078591 (90.75%) | 38920715 (70.53%) |
|  |  | 3 | 25209656 | 25208096 | 57704669 | 52118065 (90.32%) | 40298004 (69.83%) |
|  | 12 h | 1 | 25775089 | 25773145 | 58847639 | 47943103 (81.47%) | 36373355 (61.81%) |
|  |  | 2 | 26238415 | 26234509 | 59522075 | 46740444 (78.53%) | 35534824 (59.7%) |
|  |  | 3 | 21041113 | 21039823 | 47658356 | 37751045 (79.21%) | 28845252 (60.53%) |
|  | 24 h | 1 | 25511437 | 25509778 | 60909958 | 46709573 (76.69%) | 32071117 (52.65%) |
|  |  | 2 | 29645392 | 29644037 | 64285800 | 35177477 (54.72%) | 27181783 (42.28%) |
|  |  | 3 | 29961266 | 29955288 | 65823477 | 40065325 (60.87%) | 30672950 (46.6%) |
|  | 48 h | 1 | 28453008 | 28451270 | 63787042 | 46645729 (73.13%) | 35653122 (55.89%) |
|  |  | 2 | 31586250 | 31584427 | 70649101 | 48325644 (68.4%) | 36526357 (51.7%) |
|  |  | 3 | 23821687 | 23820290 | 5321642 | 36712381 (68.99%) | 27871530 (52.37%) |
| ***P. flaviflorum*** | Control | 1 | 26769834 | 26768398 | 58282818 | 33949607 (58.25%) | 26303116 (45.13%) |
|  |  | 2 | 27908329 | 27906767 | 61137346 | 37611280 (61.52%) | 29052730 (47.52%) |
|  |  | 3 | 24993513 | 24992164 | 54374491 | 32607411 (59.97%) | 25479035 (46.86%) |
|  | 0 h | 1 | 29535327 | 29533810 | 64380136 | 40894338 (63.52%) | 32168613 (49.97%) |
|  |  | 2 | 29889759 | 29888275 | 65540137 | 41866587 (63.88%) | 32496207 (49.58%) |
|  |  | 3 | 21083145 | 21082173 | 45948683 | 29271095 (63.7%) | 23049994 (50.16%) |
|  | 4 h | 1 | 25464004 | 25462220 | 54799414 | 33173406 (60.54%) | 26662232 (48.65%) |
|  |  | 2 | 30298929 | 30296075 | 65990805 | 47290817 (71.66%) | 38121338 (57.77%) |
|  |  | 3 | 26577249 | 26575740 | 57756057 | 40390239 (69.93%) | 32559712 (56.37%) |
|  | 12 h | 1 | 26319003 | 26317212 | 57044383 | 34081104 (59.74%) | 26857951 (47.08%) |
|  |  | 2 | 28281504 | 28279659 | 60879758 | 32730617 (53.76%) | 25670751 (42.17%) |
|  |  | 3 | 25281797 | 25279268 | 54473625 | 31146553 (57.18%) | 24682328 (45.31%) |
|  | 24 h | 1 | 28055439 | 28053778 | 60641592 | 41266584 (68.05%) | 33549365 (55.32%) |
|  |  | 2 | 30674648 | 30672905 | 66306427 | 39103452 (58.97%) | 30929188 (46.65%) |
|  |  | 3 | 32316654 | 32314970 | 69754687 | 44602461 (63.94%) | 35939956 (51.52%) |
|  | 48 h | 1 | 30818634 | 30817050 | 66411591 | 39800734 (59.93%) | 31837841 (47.94%) |
|  |  | 2 | 28058603 | 28056916 | 61161968 | 37259048 (60.92%) | 29039942 (47.48%) |
|  |  | 3 | 24918949 | 24917774 | 53818250 | 32721546 (60.8%) | 26092913 (48.48%) |
