# Supplementary material for: Comparative Transcriptome and Metabolome Analysis of Resistant and Susceptible Piper Species Upon Infection by the Oomycete Phytophthora Capsici
Source: Front Plant Sci. 2022 Jun 23;13:864927. doi: 10.3389/fpls.2022.864927 (PMC9278165; doi:10.3389/fpls.2022.864927)
Supplement: Supplementary Table S5 — Details of differentially expressed profiles (clusters) including genes involved in the lignin synthetic pathway. [file Table_5.DOCX]

**Table S5. Details of differentially expressed profiles (clusters) including genes involved in the lignin synthetic pathway**

| GENE ID | Times | | | | | | | | | | Gene Name | Cluster |
| --- | --- | --- | --- | --- | --- | --- | --- | --- | --- | --- | --- | --- |
|  | 0 h | 4 h | 12 h | 24 h | 48 h | 0 h | 4 h | 12 h | 24 h | 48 h |  |  |
| Pn28.318 | 246.1549 | 236.7759 | 129.7838 | 81.42838 | 130.948 | 69.07213 | 99.84821 | 57.26475 | 88.08186 | 82.10351 | PAL2 | 6 |
| Pn8.311 | 45.17454 | 8.544425 | 38.23119 | 10.48684 | 15.02832 | 2.363354 | 1.257128 | 2.513096 | 10.51872 | 7.389384 | PAL4 | 6 |
| Pn2.1269 | 74.64393 | 36.72562 | 73.53247 | 39.87425 | 51.36956 | 7.408595 | 5.499905 | 5.676331 | 16.97856 | 18.53413 | PAL4 | 4 |
| Pn8.2617 | 110.5042 | 166.055 | 128.342 | 184.0569 | 108.6984 | 64.42342 | 85.66141 | 60.09203 | 76.13554 | 70.88977 | PAL4 | 1 |
| Pn11.26 | 13.82187 | 9.799692 | 4.896978 | 5.681186 | 3.613204 | 1.583351 | 1.22646 | 0.607748 | 2.900531 | 1.975414 | C4H | 6 |
| Pn9.1366 | 3.266617 | 2.876114 | 3.091487 | 3.91234 | 3.132486 | 0.264438 | 0.121207 | 0.293751 | 0.081653 | 0.345239 | C4H | 4 |
| Pn17.1441 | 268.4217 | 194.9705 | 177.8627 | 120.2351 | 116.9504 | 101.2712 | 78.00897 | 82.7043 | 100.3803 | 110.6177 | HCT | 6 |
| Pn33.144 | 30.66402 | 26.19304 | 22.51402 | 14.83471 | 22.49781 | 12.85032 | 11.93546 | 19.28324 | 13.91254 | 13.28056 | HCT | 6 |
| Pn12.1192 | 1.427626 | 2.560134 | 4.681927 | 6.850103 | 1.048894 | 0.718744 | 1.659168 | 1.14636 | 0.544574 | 1.00777 | HCT | 1 |
| Pn23.130 | 4.754446 | 6.992289 | 7.620454 | 9.937863 | 6.861725 | 3.712565 | 0.691054 | 1.365033 | 2.211454 | 5.772498 | HCT | 7 |
| Pn16.733 | 0.668775 | 1.097488 | 0.943007 | 0.921638 | 2.857591 | 0.110062 | 0.091532 | 0.25117 | 0.337197 | 0.235902 | HCT | 3 |
| Pn16.738 | 6.316033 | 11.29697 | 5.188337 | 0.414959 | 18.63783 | 0.700462 | 1.142134 | 0.844347 | 0.54291 | 1.046273 | HCT | 3 |
| Pn15.461 | 0.852247 | 0.563312 | 0.437885 | 0.479982 | 0.41973 | 22.52016 | 8.966602 | 14.81626 | 5.946123 | 11.68341 | HCT | 2 |
| Pn33.142 | 15.86212 | 16.91416 | 14.9151 | 9.165031 | 10.84751 | 23.72317 | 21.75805 | 33.50547 | 27.34446 | 29.75062 | HCT | 8 |
| Pn41.193 | 135.7408 | 167.6929 | 144.2997 | 218.4055 | 230.0385 | 64.24986 | 78.59127 | 98.41189 | 119.1917 | 112.7599 | C3H | 7 |
| Pn11.885 | 10.23136 | 9.563804 | 15.55143 | 4.308796 | 6.208428 | 3.024872 | 1.214722 | 2.818436 | 1.848304 | 3.09231 | CCoAOMT1 | 4 |
| Pn9.205 | 110.9288 | 118.526 | 92.43448 | 119.009 | 100.761 | 146.5259 | 65.22874 | 60.99244 | 62.32957 | 7.899155 | CCoAOMT1 | 4 |
| Pn2.967 | 0.263826 | 0.203147 | 0.217287 | 0.334132 | 0.566614 | 4.400424 | 2.236621 | 2.457447 | 1.359606 | 1.111755 | CCoAOMT1 | 2 |
| Pn2.848 | 0.248254 | 0.135378 | 0.352373 | 0.353741 | 0.490202 | 1.886423 | 7.460541 | 11.20909 | 11.36639 | 11.88774 | CCoAOMT1 | 5 |
| Pn21.187 | 6.744801 | 19.91007 | 5.760766 | 14.39422 | 12.62403 | 0.371085 | 1.467067 | 2.097087 | 2.202344 | 2.001483 | F5H | 3 |
| Pn20.1025 | 2.972794 | 3.393586 | 4.379039 | 1.900722 | 2.899041 | 5.03629 | 4.254792 | 6.804675 | 6.171388 | 8.877704 | F5H | 5 |
| Pn8.2226 | 26.32167 | 16.83603 | 17.44451 | 15.44959 | 15.87712 | 3.075248 | 3.177348 | 3.500859 | 3.703987 | 5.169321 | 4CL | 4 |
| Pn10.216 | 98.61495 | 136.0239 | 90.7861 | 117.3286 | 91.47146 | 60.08961 | 70.0546 | 49.38785 | 56.0034 | 56.05855 | 4CL | 1 |
| Pn8.1406 | 4.59837 | 7.150987 | 4.60368 | 4.577507 | 8.850379 | 0.510639 | 1.969542 | 0.811023 | 1.856445 | 1.946958 | CAD | 4 |
| Pn11.236 | 15.367 | 27.39468 | 28.71336 | 37.95536 | 36.88973 | 38.13239 | 50.30225 | 46.58646 | 43.62478 | 44.63967 | CCR | 5 |
