# Supplementary material for: Comparative Transcriptome and Metabolome Analysis of Resistant and Susceptible Piper Species Upon Infection by the Oomycete Phytophthora Capsici
Source: Front Plant Sci. 2022 Jun 23;13:864927. doi: 10.3389/fpls.2022.864927 (PMC9278165; doi:10.3389/fpls.2022.864927)
Supplement: Supplementary Table S6 — Primers used for qPCR. [file Table_6.DOCX]

**Table S6 Primers used for qPCR**

| Gene | Primers name | Primers | TM of product(℃) | Length of product（bp） | The primer pair efficiencies (%) |
| --- | --- | --- | --- | --- | --- |
| PUB1 | PUB1 F | TTACCAGGACTCAGCAGCGAATG | 58.06 | 120 | 96.34 |
|  | PUB1 R | AAGCCAATGACTTTACATCCTCCAG | 58.97 |  |  |
| PN11.246 | CCOAOMT F | TCTACACCGGCTACTCCCT | 58.99 | 116 | 93.75 |
|  | CCOAOMT R | CTTCTCTATGACCGGCAAGC | 58.42 |  |  |
| PN22.496 | CAD1 F | TTCGGGAAGCGTTTTGTGAG | 59.06 | 128 | 90.23 |
|  | CAD1 R | TGGTTGATGTAGTCGGGCTT | 59.02 |  |  |
| PN18.429 | CHS3 F | CCAGCTACAACCACATCGAC | 58.64 | 143 | 90.10 |
|  | CHS3 R | TGGGATGATTGTTTGCGACG | 59.20 |  |  |
| PN24.640 | 4CL1 F | TGTCTTCCCCAACGATCCAA | 58.94 | 104 | 90.04 |
|  | 4CL1 R | ACTCCCCAGAAACAGCATCA | 58.93 |  |  |
| PN17.616 | 4CL2 F | CTTGTACTCCTCTGGCACCA | 59.02 | 146 | 91.10 |
|  | 4CL2 R | GGAACATGGGCAGGAAACAA | 58.66 |  |  |
| PN16.1237 | 4CL3 F | CGGTGAAGTTCCCATTGCTT | 58.75 | 148 | 92.30 |
|  | 4CL3 R | TGAAGCTGATTTGGGCACAC | 59.04 |  |  |
| PN8.2226 | 4CL4 F | TGGGAAGGAGCTTGAAGAGG | 59.01 | 104 | 91.29 |
|  | 4CL4 R | ACAGGCTCATGGTCAGAACT | 58.64 |  |  |
| PN12.1639 | CCR1 F | GACTGCCCATCTGAGGAAGT | 59.09 | 114 | 90.99 |
|  | CCR1 R | AGAACTCCAACACAGCCAGT | 59.16 |  |  |
| PN12.1104 | CCR2 F | TCATTTCAGTGACTTCGCCG | 58.57 | 107 | 91.21 |
|  | CCR2 R | TCGGATTACTGGCCCTTACC | 58.87 |  |  |
| PN17.912 | CCR3 F | AGCAGCTCCCTTACGATGAA | 58.80 | 140 | 91.19 |
|  | CCR3 R | GTCCTCCCTCCAGATTACGG | 58.96 |  |  |
| PN8.311 | PAL1 F | TGTGAGGTGATGAACGGGAA | 58.95 | 132 | 92.23 |
|  | PAL1 R | GGCGGCTTTCATGTAGGAAC | 59.27 |  |  |
| PN8.2617 | PAL2 F | TGATGATCCTTGCAGCTCCA | 59.09 | 100 | 91.26 |
|  | PAL2 R | GTTGCCCTCCTTCTCCTTCT | 59.01 |  |  |
| PN28.318 | PAL3 F | GAAGGACCCATTGACGAAGC | 58.91 | 146 | 93.12 |
|  | PAL3 R | CGATGAGTGGGTTGTCGTTC | 58.93 |  |  |
| Pn10.2166 | JMT F | GGAGAGATGAGCTACGCAAAG | 58.53 | 150 | 90.12 |
|  | JMT R | ACCCAAGTCCGCCATGATTA | 59.08 |  |  |
| Pn11.2177 | ARF19 F | TCAGCAGGCATCAACTCTCA | 59.02 | 123 | 90.10 |
|  | ARF19 R | TTGCTGACTTGACCACATGC | 59.05 |  |  |
| Pn15.1461 | COI1 F | GGAGGAGAGTAGCGTCTTGG | 59.26 | 138 | 91.13 |
|  | COI1 R | TTTGCAAGCAGTTCCAGGTC | 58.97 |  |  |
| Pn16.844 | ACO1 F | ATTGATGGGTTGGGTGTTGC | 59.02 | 101 | 91.09 |
|  | ACO1 R | CCAACAACTCCAGGCAGAAC | 59.05 |  |  |
| Pn17.934 | MYC2 F | GGTGCAGATACTAGGGAGGG | 58.66 | 142 | 92.12 |
|  | MYC2 R | CATCAACTCGTTCACCACCG | 59.21 |  |  |
| Pn30.296 | ETR1 F | CGAACTCCAATGGACGCAAT | 58.91 | 117 | 91.04 |
|  | ETR1 R | TGTGGAAAGAAGGTTGCTGC | 58.97 |  |  |
| Pn33.279 | ERF1 F | GTGAAGGTGGAGTTGGAGGA | 58.94 | 109 | 90.99 |
|  | ERF1 R | AACTTGTTGGAACGCTGCAT | 58.97 |  |  |
| Pn3.621 | NPR1 F | TGGGCATGGAAGTCTTGGAT | 59.00 | 116 | 90.10 |
|  | NPR1 R | CCAGTGTAGTATGCCCCTCC | 59.24 |  |  |
| Pn3.650 | GA3 F | CGAATGGGCATTGTTTGAGC | 58.37 | 107 | 90.12 |
|  | GA3 R | GCAAGTGGTCCTCTGTCAAC | 58.77 |  |  |
| Pn37.123 | ABA2 F | AAGGTTGGAAGGGAAGACGG | 59.60 | 104 | 91.24 |
|  | ABA2 R | CAACGATGAACACCTTGGCG | 60.11 |  |  |
| Pn39.35 | JAZ F | AGCAACTTCAAGCAGACGTG | 59.06 | 141 | 91.35 |
|  | JAZ R | TCTTGGGCAGCAAATCCATC | 58.52 |  |  |
| Pn4.2277 | PAD4 F | CGGTGCTTGGTGAAGACAAA | 58.98 | 120 | 92.34 |
|  | PAD4 R | TGGGAAGAATGGGTTTGCAC | 58.66 |  |  |
| Pn7.1179 | AUX1 F | TGGTCCTATACTTCACCGGC | 58.88 | 100 | 90.17 |
|  | AUX1 R | AACTTTTGCGGCTTCCACAT | 58.96 |  |  |
| Pn8.814 | NPR3 F | TTCCTCACTGCTCCCAAGTT | 58.86 | 129 | 93.14 |
|  | NPR3 R | CTTCCCGTTTCAGCTCACAG | 58.85 |  |  |
